# Potential core species and satellite species in the bacterial community within the rabbit caecum

**DOI:** 10.1111/j.1574-6941.2008.00611.x

**Published:** 2008-11

**Authors:** Valérie Monteils, Laurent Cauquil, Sylvie Combes, Jean-Jacques Godon, Thierry Gidenne

**Affiliations:** 1 INRA, Université de Toulouse, UMR 1289, Tissus Animaux, Nutrition, Digestion, Ecosystème et Métabolisme, Chemin de Borde-Rouge, AuzevilleCastanet-Tolosan, France; 2 INPT-ENSATCastanet-Tolosan, France; 3 ENVTToulouse, France; 4 INRA, UR 050, Laboratoire de Biotechnologie de l'EnvironnementNarbonne, France

**Keywords:** digestive ecosystem, caecum, bacteria, molecular library, rabbit, phylogeny

## Abstract

A bacteria library was constructed from the caecum of a rabbit maintained under standard conditions. The complete gene 16S rRNA gene was sequenced. The 228 clones obtained were distributed in 70 operational taxonomic units (OTUs). The large majority of the OTUs were composed of one or two clones and seven OTUs contained half of the sequences. Fourteen sequences had high similarity to the sequence already registered in databases (threshold of 97%). Only one of these sequences has been identified as *Variovorax* sp. (99% identity). Units were distributed mainly (94%) in the *Firmicutes* phylum. Three sequences were related to *Bacteroidetes*. Nine clusters were defined in the phylogenic tree. A great diversity of caecal bacteria of the rabbit was shown. Half of the sequences generated in this library were distributed in the phylogenetic tree near the sequences characterized previously in rabbit caecum (potential core species), and the other half of the sequences were well separated (satellite species).

## Introduction

In rabbit breeding, the incidence of digestive troubles after weaning is high, leading to a relatively high mortality rate (*c*. 10% in France, Azard, 2006) in spite of preventative antibiotherapy. The caecal ecosystem is supposed to play a key role in the digestive health of the young rabbit (Gidenne, 2003; Gidenne *et al.*, 2004). Few studies have characterized the caecal microbiota using molecular tools. For adult humans, the faecal flora was shown to be unique for each person and the dominant active flora stable over time (Zoetendal *et al.*, 1998). For cows, time stability of the digestive flora was also observed (Martin *et al.*, 2001). The hypothesis of stability of flora in the adult rabbit caecum could be made and reinforced by the low incidence of digestive troubles after the critical period of weaning. Previous studies on rabbit caecal microbiota revealed that bacterial species are mainly strictly anaerobic, but they were mostly performed using classical culture-based techniques. Boulahrouf *et al.* (1991) identified *Eubacterium cellulosolvens* and *Bacteroides* spp. as the predominant species in a 58-day-old rabbit. More recently, a molecular approach has allowed study of species even if no information is known about the necessary culture conditions. Using oligonucleotide probes, other species were found in caecal content in 70-day-old animals: *Fibrobacter succinogenes, Fibrobacter intestinalis, Ruminococcus flavefaciens* and *Ruminococcus albus* (Bennegadi *et al.*, 2003). A single library of caecal bacteria was characterized by Abecia *et al.* (2005) with rabbits (56 days old) and showed numerous novel sequences of bacteria. However, a more detailed bacterial inventory is necessary as a first step to characterize the caecal microbiota. Thus, it is important to be as exhaustive as possible, in order to be representative of the diversity of the community, including low-abundant species. We chose to sequence numerous clones from one individual sample, rather than to study a pool of several samples because of the dilution effect.

Therefore, this work presents a library based on the sequencing of the complete gene of 16S rRNA of the caecal bacteria population for an adult rabbit maintained in a standardized physiological state. We aimed to precisely characterize the whole bacterial community on an animal without causing any disturbances and on an individual sample to inventory a maximum of bacterial species present.

## Materials and methods

A group of 12 adult rabbits (INRA 1067 × PS HYPLUS 79, Grimaud, France) were reared in a controlled environment (housing, diet and prophylaxis), at the Institut National de la Recherche Agronomique (INRA TANDEM, Toulouse, France). Rabbits were kept in individual metabolism cages (55 × 40 cm) and exposed to a 12-h light (07:00–19:00 hours) and 12-h dark schedule. After weaning (35 days) and until the end of the trial (7 months old, 4.65 kg), rabbits were fed a commercial standard pelleted diet (without coccidiostatic and without antibiotic). The diet was provided twice a day and was composed of wheat bran, sunflower meal, beet pulps, wheat, soya, alfalfa, barley, sodium chloride and calcium carbonate. The chemical composition of the pellets was 44.2% neutral detergent fibers, 30.8% acid detergent fibers, 8.3% acid detergent lignin, 22.5% cellulose, 13.4% hemicelluloses, 18.9% crude protein, 8.4% minerals and 2% fat matter. The distributed quantity (80% of the intake registered the month before the slaughter) was defined in order to ensure a total and constant daily intake (207 g day^−1^). Water was provided with *ad libitum* access. One rabbit among the 12 was chosen for the library fulfulling the criteria of regular intake, weight at the average of the group and no digestive disturbances. We chose to study one rabbit to detect minor species and avoid loss of information. A pool of several samples, before or after DNA extraction, involves dilution of species of low abundance into the community.

Caecal content was collected after Imalgene (Imalgene®, Rhône Merieux, France) anaesthesia was injected into the intramuscular region (1 mL) and T61® euthanasia in the endocardiac (1.5 mL) region. The rabbit caecum was reached through a small midline incision (5–8 cm) on the abdomen. The caecal content was immediately sampled and stored at −80 °C. DNA was extracted from frozen samples using a QIAamp DNA Stool Mini Kit (QIAGEN Ltd, West Sussex, England) following the manufacturer's instructions. DNA extracts were stored at −20 °C.

The amplification of 16S rRNA gene was carried out as described by Godon *et al.* (1997) and Duthoit *et al.* (2003) with the universal primer W02 (GNTACCTTGTTACGACTT, corresponding to the *Escherichia coli* position 1509) and the bacterial primer W18 (GAGTTTGATCMTGGCTCAG, corresponding to the *E. coli* position 9). The complete gene of 16S rRNA was obtained (1500 pb). PCR was performed using the following program: 2 min of initial denaturation at 94 °C, followed by 25 cycles of denaturation (1 min at 94 °C), annealing (1 min at 50 °C) and extension (1 min at 72 °C), with a final extension at 72 °C for 10 min. PCR products were purified on QIAquick PCR purification kit columns (QIAGEN). The size of the PCR products was verified with an electrophoresis (0.8% agarose gel) migration, followed by ethidium bromide staining for viewing. PCR products were ligated into a pCR4-TOPO and transformed into *E. coli* TOP 10 One Shot as specified by the manufacturer (Invitrogen). Transformed clones were selected and grown overnight in Luria–Bertani agar plates with kanamycin selection (25 mg mL^−1^). Plasmid DNA was isolated from 293 selected transformed clones using the Montage Plasmid Miniprep 96 kit (Millipore) following the manufacturer's instructions. DNA inserts were sequenced at the Centre de Ressources Genotypage Sequençage, platform of Toulouse (France). The 16S rRNA gene sequences were sequenced bidirectionally using the ABI Prism Big Dye Terminator on an ABI DNA sequencer 3730. The sequences obtained were assigned to the operational taxonomic unit (OTU) using a threshold of 97% similarity of sequence. Each OTU was represented by the longest sequence. A rarefaction curve was produced using analytic rarefaction 1.3, which is available online (http://www.uga.edu/~strata/software/index.html). The coverage of the clone library was calculated according to the formula of Good (1953). For each OTU, the longest sequence was compared with sequences available in the NCBI database using blastn.

The phylogenetic analysis was based on the aligned homologous nucleotides. To construct the phylogenetic tree, sequences were aligned automatically to the *E. coli* in the database SSURef_02_07_1_4_tree_silva_opt generated by the SILVA project (Pruesse *et al.*, 2007) using the fastaligner tool in the ARB sequence environment. The sequences were integrated into the corresponding tree using a maximum parsimony method (Ludwig *et al.*, 2004). The tree was determined by repeating the process by bootstrapping (1000 iterations were performed).

The nucleotide sequence data have been deposited in the GenBank database with accession numbers EF445140–EF445209.

The library was compared with the previously published data about the rabbit's caecum, using unifrac analysis (Lozupone *et al.*, 2006). This project is available online (http://bmf2.colorado.edu/unifrac/index.psp). The phylogenetic diversities and the effect of the environment on the diversity were compared. A lineage-specific analysis was performed to determine the contributions of the environments if they were significantly different.

## Results

**Fig. 1:**
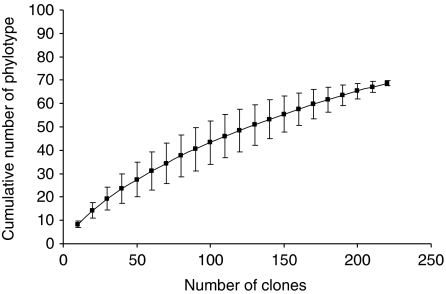
Rarefaction curve generated for the 16S rRNA gene in the bacterial library (228 clones) from adult rabbit caecum with analytic rarefaction 1.3 software. The different clones were grouped into OTUs at a level of sequence identity ≥97%. Error bars indicated 95% confidence intervals.

The complete sequences (228) were distributed in 70 distinct OTUs with a threshold of 97% sequence similarity to define an OTU (Table 1). Half of the sequences were represented by only seven OTUs (from 8 to 32 sequences within an OTU). The majority of OTUs (53 of 70) were composed of one or two sequences. The coverage of the clone library was 84% for the 228 clones. A low decrease in the rate of OTU detection could be observed with the rarefaction curve (Fig. 1).

**Table 1:** Distribution of the 228 clones within the 70 OTUs (with identity cut-off 97% between the sequences of clones) according to the similarity of sequence with the NCBI database

OTU name	% of similarity*	Nearest sequence in the NCBI database		Origin of the nearest sequence	Number of clones in the OTUs	Cluster
NED1D3	99	UB	DQ905060	Human faeces	2	VI
NED2D4	99	UB	AY993615	Mouse caecum	2	IV
NED2F10	99	UB	DQ777919	Rat faeces	2	VI
NED1B6	99	*Variovorax sp.*	AB196432	Soil	1	I
NED1E5	98	UB	AJ863539	Rabbit caecum	13	IV
NED2D1	98	UB	AB264069	Dugong faeces	2	II
NED2F5	97	UB	DQ824540	Human faeces	10	VI
NED2A9	97	UB	DQ815741	Mouse caecum	7	VII
NED1C12	97	UB	DQ394667	Reindeer rumen	2	VII
NED1E3	97	UB	DQ815580	Mouse caecum	1	IV
NED2G1	97	UB	DQ394637	Reindeer rumen	1	VII
NED3C7	97	UB	AB270018	Heifer rumen	1	VII
NED3G6	97	UB	DQ456201	Turkey caecum	1	VII
NED3H1	97	UB	DQ824540	Human faeces	1	VI
NED2A2	96	UB	DQ815454	Mouse caecum	3	V
NED2F7	96	UB	AY916320	Human stool	2	VI
NED2B8	96	UB	AF371819	Swine intestine	1	VII
NED3F5	96	UB	AY854288	Herbivore gastrointestine tract	1	VI
NED3B6	95	UB	DQ456150	Turkey caecum	7	VII
NED2E8	95	UB	DQ777932	Rat faeces	3	IV
NED1H5	95	UB	DQ815594	Mouse caecum	2	VI
NED1H7	95	UB	DQ455958	Turkey caecum	2	V
NED2C2	95	UB	AJ863536	Rabbit caecum	2	IV
NED2F1	95	UB	DQ815887	Mouse caecum	2	VI
NED1D11	95	UB	DQ815739	Mouse caecum	1	VI
NED2H8	95	UB	DQ777935	Rat faeces	1	IV
NED3A12	95	UB	DQ815738	Mouse caecum	1	VI
NED3A4	95	UB	AB009189	Bovine rumen	1	VII
NED3A9	95	UB	DQ905367	Human faeces	1	VII
NED3G12	95	UB	AF132261	Human faeces	1	VI
NED2H6	94	UB	AB185589	Cattle rumen	8	VI
NED3A6	94	UB	AB185589	Cattle rumen	3	VI
NED2C12	94	UB	AF371829	Swine intestine	2	VI
NED2E3	94	UB	AF371824	Swine intestine	2	VI
NED3B2	94	UB	AF371654	Swine intestine	2	IV
NED1B8	94	UB	AJ408989	Human large intestine	1	VI
NED1C7	94	UB	DQ808697	Human faeces	1	V
NED2B3	94	UB	DQ014691	Mouse caecum	1	VI
NED2D10	94	UB	DQ673492	Rumen	1	IX
NED2E6	94	UB	DQ806041	Human faeces	1	VI
NED3B3	94	UB	DQ815486	Mouse caecum	1	IV
NED3H5	94	UB	DQ905260	Human faeces	1	VII
NED3G11	93	UB	DQ673521	Rumen	15	IV
NED1D5	93	UB	EF098126	Mouse caecum	3	IV
NED1G12	93	UB	DQ904637	Human faeces	2	VIII
NED1B12	93	UB	DQ905458	Human faeces	1	VII
NED1C3	93	UB	AF371820	Swine intestine	1	VII
NED1F8	93	UB	DQ815741	Mouse caecum	1	VII
NED1H12	93	UB	DQ815741	Mouse caecum	1	VII
NED1H8	93	UB	DQ801345	Human faeces	1	IV
NED2A6	93	UB	EF099149	Mouse caecum	1	IV
NED3B1	93	UB	AB237713	Sedimentary rock milieu	1	IV
NED3E1	93	UB	EF097618	Mouse caecum	1	II
NED2B11	92	UB	DQ057368	Broiler chicken ileum and caecum	28	IV
NED2A3	92	UB	DQ815486	Mouse caecum	5	IV
NED2E5	92	UB	CT574154	Wastewater treatment	4	II
NED3D6	92	UB	AB234499	Termite gut	2	VI
NED1B3	92	UB	DQ815486	Mouse caecum	1	IV
NED2C11	92	UB	DQ673521	Rumen	1	IV
NED2H3	92	UB	DQ441345	Human intestine biopsy	1	VI
NED3B11	92	UB	DQ824886	Human faeces	1	IV
NED1G7	91	UB	AY986384	Human stool	32	VI
NED3C2	91	UB	AB264081	Dugong faeces	9	III
NED2G5	91	UB	AB269988	Cow rumen	5	VI
NED3D4	91	UB	AF371530	Swine intestine	2	IX
NED1F6	91	UB	DQ809218	Human faeces	1	IX
NED3E10	90	UB	AF371777	Swine intestine	3	VI
NED1A12	90	UB	AB009176	Rumen	2	VI
NED1B5	90	UB	EF096987	Mouse caecum	1	IV
NED1A10	89	UB	AB009232	Rumen	1	VI

**Fig. 2:**
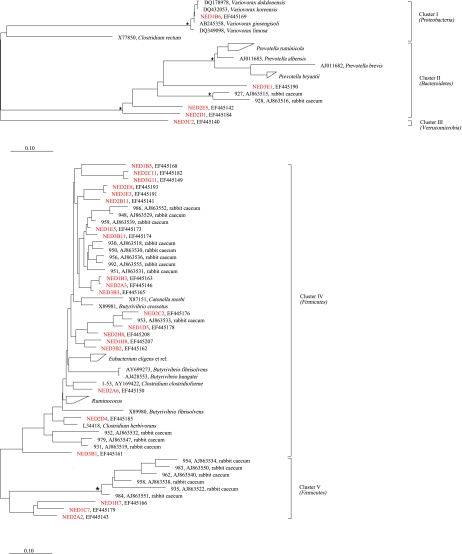

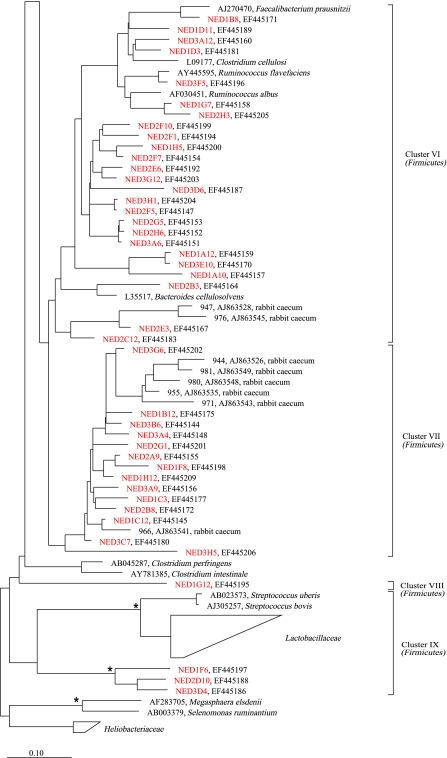
Phylogeny placement of total 16S rRNA gene sequence data recovered from the caecum of an adult rabbit for bacteria. The tree was constructed using the SILVA project and the ARB sequence environment using a maximum parsimony method. Bootstrap values derived from 1000 iterations; only the values <90% are marked with an asterisk. Scale bar represents the number of substitutions per nucleotide position.

The nearest sequence in the database and their environment origin were mentioned. A cross-reference to the phylogenetic tree presented in Fig. 2 was made with the cluster number.

* Percentage similarity between the longest sequence of each OTU generated in our library and the sequences available in the NCBI database.

UB, uncultured bacteria.

The blast data indicated that among the 70 OTUs, 56 of the OTUs did not correspond to any recorded entries in the NCBI database. These sequences can be considered as novel sequences with an identity of <97% with the sequences of the database. The 14 other sequences had 97% or more identity with an already characterized sequence (Table 1). A single clone (NED1B6) had a high identity (99%) with a cultured species, *Variovorax* sp. from the *Proteobacteria* phylum. All the other 13 clones among these had a high identity with the uncultured bacteria. Except for *Variovorax* sp., which was obtained from a soil sample, all the sequences related to ours with a high similarity and have digestive origins from different areas of the gastrointestinal tract of ruminal or monogastric animals. For the OTU with lower identity of sequence with the database, the nearest sequences were also of digestive origin, except for two OTUs (NED3B1 and NED2E5). Sixty-five OTUs were distributed in the *Firmicutes* phylum. The five remaining OTUs were distributed into three phyla: three OTUs in *Bacteroidetes*, one OTU in *Proteobacteria* and one OTU in *Verrucomicrobiae*.

The distribution in a phylogenetic tree of sequences generated from this study is shown in Fig. 2. The pintail values were good because >90% of the sequences had an associated pintail value ≥90. The great majority of bootstrap values were >90% (data not shown). Only eight bootstrap values were <90% (marked with asterisks in Fig. 2). Among these, seven values ranged from 70% to 90% and the lowest value was 58%, which was still considered a good value. The 70 sequences of this library cluster into nine groups as indicated in the tree (Fig. 2). A first dichotomy in the tree divided groups I, II, III, and others clearly.

Cluster I contained a single sequence (NED1B6) within the *Proteobacteria* phylum. This sequence was the only one with high identity (99%) with the cultivated species, *Variovorax* sp. The branches were very short, and the bootstrap values were strong (100). These data indicated a high identity in this group.

Three sequences were related to cluster II and the *Bacteroidetes* phylum, including two novel sequences (NED3E1 and NED2E5) and one sequence of uncultured bacteria characterized previously (NED2D1, Table 1). Two other sequences observed by Abecia *et al.* (2005) in the caecum of a growing rabbit were also distributed in this cluster, but the identity between sequences was lower than the threshold of 97%. Several characterized species were related to this group: *Prevotella ruminicola, Prevotella albensis, Prevotella brevis* and *Prevotella bryantii*. However, the high lengths of branches showed clearly a low identity within the group.

Cluster III was represented by a single novel sequence (NED3C2) in the *Verrucomicrobia* phylum. This group was clearly different from the latter two clusters (bootstrap value of 99%).

The clusters IV–IX were included in the *Firmicutes* phylum. Nineteen sequences were affiliated to cluster IV, of which three sequences were already characterized (NED1E3, NED1E5 and NED2D4, Table 1) and 16 were novel sequences. Cluster IV represented the second main cluster, with 83 clones in this library. All sequences could be considered as close because of the very short branches of the tree. Three sequences had high identity with sequences characterized from the caecum of the rabbit or the mouse. This cluster held 12 other sequences obtained from the other rabbit caecum library, as well as *Butyrivibrio fibrisolvens* and *Ruminococcus* spp.

Cluster V contained three novel sequences that stemmed from our study and six sequences of caecum rabbit stemmed from Abecia's library (2005). Within this cluster, the sequences generated from our study were well distinguished from the sequences characterized in the other library. Two subclusters could be defined and were reinforced by the long branches. This cluster is not related to any isolates described previously.

Twenty-five sequences were related to cluster VI with 21 novel sequences and four sequences (NED1D3, NED2F10, NED3H1 and NED2F5) characterized previously in monogastric faeces (human or rat, Table 1). This group represented the main part of our library (89 clones). Two other sequences of uncultured bacteria cloned from rabbit caecum (Abecia *et al.*, 2005) were placed in this group. *Ruminococcus flavefaciens, R. albus* and *Bacteroides cellulosolvens* were also related to the cluster VI.

Among the 14 sequences distributed in cluster VII, nine sequences were novel and five were already observed in the caecum or the rumen of different species (NED3G6, NED2G1, NED2A9, NED1C12 and NED3C7, Table 1). Five sequences that were obtained previously from the rabbit caecum were also placed in this group. Group VII was close to cluster VI but well separated from it.

A single novel sequence was related to cluster VIII (NED1G12). This group was clearly distinguished from clusters IV, V, VI and VII even if all these groups had a common branch at the base of the tree.

The final cluster (cluster IX) contained three novel sequences (NED1F6, NED2D10 and NED3D4). These sequences were relatively closely related to each other. *Streptococcus uberis, Streptococcus bovis* and *Lactobacillaceae* were related to this group, but were rather far away from the sequences of our library.

The environments of our library and the previously published library of Abecia *et al.* (2005) shared 13% of the total phylogenetic diversity contained by both together (unifrac analysis, data not shown). The pattern of environments was significantly different (*P* <0.01). Among the nodes in the phylogenetic tree, only one node of cluster V contributed significantly (*P* <0.01) to the differences between environments. This significant node was the one that separated, within cluster V, the sequences that stemmed from the Abecia library and the library presented in this paper.

## Discussion

The distribution of clones within OTU showed five OTUs holding 10 or more clones. The great majority of OTUs (75%) contained one or two clones. These results demonstrated the great diversity of the rabbit caecal ecosystem. A large majority of OTUs with only one sequence were also observed in the gut tract of other herbivorous animals such as equine large intestine (Daly *et al.*, 2001) or cow rumen (Tajima *et al.*, 2007). Herbivorous digestive ecosystems appear to have a strong diversity. The weak percentage (13%) of diversity shared between the two libraries available for the rabbit caecum reinforced these data. However, the coverage of the clone library was high (84%), meaning that the major part of the diversity in the library had been detected. In this library, the main part of the 70 OTUs corresponded to new sequences with 56 novel sequences and 14 sequences having high identity with clones sequenced previously (identity cut-off 97%). Only one sequence had >97% similarity to a cultured species, *Variovorax* sp., identified in a soil ecosystem. All other sequences (69 sequences i.e. novel sequences and sequences related to already characterized sequences) corresponded to uncultured bacteria. All sequences (except *Variovorax* sp.), having high identity with sequences registered in the database, originated from digestive ecosystems. The related sequences had been identified along the digestive tract (rumen, caecum and faeces) of monogastric (human, mouse and turkey), ruminant (reindeer and heifer) or nonruminant herbivorous animals (dugong and rabbit). There was a great diversity of hosts but a common digestive ecosystem origin. Besides being herbivores, the rabbit is a caecotrophic animal, excreting two types of faeces, soft and hard, and ingesting only the soft one. This behaviour improves the digestive efficiency for proteins and fibres through a valorization of microbial protein of soft faeces. Therefore, the caecal ecosystem plays a key role in the rabbit digestive physiology, because of its size (40% of the whole tract content) and of the highly active microbiota. We hypothesized that the rabbit caecal bacterial community was close to other herbivorous animals like ruminants. Nevertheless, this study demonstrated that among the OTUs that had high identity rates (>97%), a majority of sequences were characterized in monogastric animals and not in herbivorous animals. These data underline the common characteristics of digestive ecosystems from herbivorous and monogastric animals.

Three of these OTUs had high identity scores (98%, 97% and 97%, respectively, for NED1E5 in cluster IV, NED2F5 in cluster VI and NED2A9 in cluster VII) and represented high numbers of clones (13, 10 and 7, respectively). Nevertheless, the two main OTUs (32 and 28 clones) were never found in other studies even if they represent >25% of the clones of this library. The molecular profiling performed was relatively complete because the coverage rate indicated a probability of 16% for obtaining a new sequence if a supplementary clone was sequenced at random. Conversely, both available libraries for the rabbit caecum presented a weak value of shared diversity, suggesting that within the rabbit species a weak part of the diversity is characterized.

The *Firmicutes* phylum was largely dominant (94%) in the bacterial flora of rabbit caecum. Among the clusters related to this phylum, clusters IV and VI were the most numerous, with 172 of 228 clones of the library. In the horse's large intestine, the *Firmicutes* phylum was known to contain the majority of cellulolytic and fibrolytic organisms (Daly *et al.*, 2001). Interestingly, the dominance of *Firmicutes* was more marked in the rabbit caecum than in the horse's large intestine (72%, Daly *et al.*, 2001) or in the rumen of the cow in fibre-associated bacteria (44%, Koike *et al.*, 2003) or in the total content (40–95%, Tajima *et al.*, 2000, 2007). Surprisingly, the largest part of the sequences related to the *Firmicutes* phylum in the rabbit caecum corresponded to the value observed in the rumen of the cow with a very high concentration level in the diet (95% of sequences related to *Firmicutes*). *Ruminococcus flavefaciens, F. succinogenes, R. albus* and *F. intestinalis* were detected in the rabbit caecum using a dot-blot hybridization technique (Bennegadi *et al.*, 2003). The phylogenic tree obtained with this library showed a sequence (NED3F5 i.e. one clone) close to *R. flavefaciens* but with an identity level <97%. These data reinforce the result observed by Abecia *et al.* (2005) in rabbit caecum. *Ruminococcus flavefaciens* was shown, using cultivation techniques, to be the predominant cellulolytic species in the horse's caecum, another herbivorous and nonruminant animal (Julliand *et al.*, 2001). No sequence of this library was related to *F. succinogenes*, whereas the dot-blot hybridization technique allowed its detection (Bennegadi *et al.*, 2003). These data agreed with the hypothesis of amplification of *F. succinogenes* being less efficient by PCR in comparison with other gut bacteria (Tajima *et al.*, 2001). *Ruminococcus albus* was detected in this library (two sequences: NED1G7 i.e. 32 clones and NED2H3 i.e. one clone) and was also observed by Bennegadi *et al.* (2003) and Abecia *et al.* (2005) in the rabbit caecum. This bacterial species was detected in all the studies on the rabbit caecum whereas no sequence fitted with *R. albus* in microbial diversity recovered in the equine gut (Daly *et al.*, 2001). This major cellulolytic bacterium was identified in the rabbit caecum and cow rumen (Tajima *et al.*, 2000), but was not present in the equine gut, where the cellulolytic activity was also essential.

In our library, three sequences (NED3E1, NED2E5 and NED2D1 i.e. seven clones in total) were related to the *Bacteroidetes* phylum. These data did not confirm the dominance of *Bacteroidetes* in rabbit digestive flora observed by Gouet & Fonty (1979) and corroborated by Boulahrouf *et al.* (1991) and Carabaño *et al.* (2006). In our study, *Bacteroidetes* represented only a small part (4%), whereas Abecia *et al.* (2005) did not detect any clone that fitted within this group. In the equine large intestine, 3% of recovered sequences were clustered with *Bacteroidetes* (Daly *et al.*, 2001). This community appeared more important in the rumen, with 35% of sequences related to this phylum (Whitford *et al.*, 1998). These results indicated that the characteristics of the rumen seem to be better adapted for *Bacteroidetes* than the caecal environment. These characteristics could have evolved widely between animal species: related to the dietary behaviour, like the intake level, the number of meals and, consequently, the intensity of postfeeding modifications and the nutrient flux; related to the anatomy of animals like the tract length, the retention time, the particle size, the saliva impregnation, the presence of different fractions in the digestive content, interaction with digestive secretions and the availability of the nutrients and their sources; or related to the physico-chemical parameters like the aerobic level, the pH range, etc. No sequenced clone was related to *Prevotella* in this study or in the previous rabbit caecum library (Abecia *et al.*, 2005). In the equine gut, *Prevotella* communities were affiliated to two novel subgroups clearly distinguished from *P. albensis* and *P. bryantii* represented in the rumen (Ramsak *et al.*, 2000). *Prevotella* present in digestive ecosystems seemed very specific to animal species with a specific group for ruminants and another group for equines. No group was identified for rabbits.

Despite the high coverage of this library and the number of clones, only one sequence (NED1E5) had >97% identity with one sequence of the available rabbit caecum library (clone 959, Abecia *et al.*, 2005). The significant difference between the patterns of environments was supported by a few similar sequences. In spite of these data, only one node in the phylogenetic tree contributed significantly to the difference between both libraries. The bootstrap value associated with this node was relatively weak (79%). These data could be surprising, but in the phylogenic tree, all sequences obtained by Abecia *et al.* (2005) were distributed within the clusters that we had defined. Cluster IV contains 12 sequences characterized by Abecia *et al.* (2005). Nine of these sequences were clustered by Abecia *et al.* (2005) in a specific rabbit group without other sequences of the database. For these authors, the caecum conditions involved some specific characteristics and allowed the development of species adapted for a caecotrophic lifestyle. Several of our sequences were close to the sequences generated by Abecia *et al.* (2005) and reinforced the hypothesis of rabbit caecum-specific species adapted to this particular biotope, with notably a short rate of passage (6–12 h) and highly anaerobic conditions (−220 mV, Kimse *et al.*, 2008). For instance, bacterial species adapted to hydrolyse quickly fermentable polysaccharides (such pectins or hemicelluloses) would be favoured in the caecal biotope, compared with cellulolytic bacteria (Carabaño *et al.*, 2006). The hypothesis of specific groups was also proposed for the horse by Daly *et al.* (2001). It could be supposed that rabbit and the equine bacterial population may be partly common, because both these animal species have a similar hindgut physiology. Unfortunately, we cannot verify this hypothesis because the equine sequences obtained by Daly *et al.* (2001) were not long enough to be included in the SILVA database (SSURef_02_07_1_4_tree_silva_opt generated by the SILVA project).

Most of the sequences generated in this library were clustered with sequences of the library of Abecia *et al.* (2005). However, several other sequences were more isolated. Looking at these data, two different categories of bacteria could be defined. The first one could correspond to the potential core species of bacterial flora in the caecum of the rabbit. This category contains the nearest sequences from the library of Abecia *et al.* (2005) and ours. A second category could correspond to satellite species containing the most isolated sequences. This part of the bacterial flora could be more sensitive to the environment than breeding conditions or an individual effect. Only one node of the phylogenetic tree explained the significant difference between libraries. However, the similarity between sequences that stemmed from both libraries was weak. This could be explained by several factors: first, the age and the breeding differences. In the study of Abecia *et al.* (2005) rabbits were fed an intensive production regime for the growth of the animal. The diet fed in our study probably had less intensive characteristics (adapted to the maintenance status of an adult rabbit) with a lower intake level. It was shown that the composition of diets had an impact on the existing flora for the rabbit (Bennegadi *et al.*, 2003) and the ruminant (Goad *et al.*, 1998; Kocherginskaya *et al.*, 2001; Koike & Kobayashi, 2001; Reilly *et al.*, 2002; Mosoni *et al.*, 2007). In addition, more clones (228 vs. 96) were sequenced in our study in comparison with the library of Abecia *et al.* (2005), and more OTUs were defined (70 vs. 44). These data could also explain the low similarity between both studies, with a higher diversity detection in our study.

In conclusion, the majority of sequences obtained in this library of rabbit caecum bacteria were novel. These data show the great bacterial diversity of the ecosystem studied. The very large majority of sequences were related to the *Firmicutes* phylum. Almost half of the sequences were placed in the phylogenic tree close to bacterial sequences identified in caecum rabbit. These data support specific characteristics for rabbits that involved a part of the flora fitted to the caecal biotope. These sequences may represent common bacterial species of caecum rabbit i.e. potential core species. However, our study shows that half of the sequences were well separated from other rabbit caecum sequences. These satellite species may be influenced more by environmental or host individual factors.
